# The Role of Multimodality Imaging in Guiding Ventricular Tachycardia Ablation: An Updated Review

**DOI:** 10.31083/RCM47045

**Published:** 2026-05-18

**Authors:** Eiad Habib, Mahmoud Abdelnabi, Ramzi Ibrahim, Christopher Kanaan, Hoang Nhat Pham, Ahmad Alkhatib, Hesham Abdalla, Hunter VanDolah, Adam Bacon, Malak Tahsin, Fares Jamal, Abdulrhman Eldeib, Reza Arsanjani, Hicham El Masry

**Affiliations:** ^1^Department of Cardiovascular Medicine, Mayo Clinic, Phoenix, AZ 85259, USA; ^2^Department of Medicine, University of Arizona, Tucson, AZ 85721, USA; ^3^Department of Cardiovascular Medicine, Mayo Clinic, Rochester, MN 55902, USA; ^4^Department of Internal Medicine, MedStar Health, Baltimore, MD 21044, USA; ^5^Department of Internal Medicine, Mayo Clinic, Phoenix, AZ 85259, USA; ^6^Department of Internal Medicine, Creighton University, Phoenix, AZ 85013, USA; ^7^Department of Hematology and Oncology, Mayo Clinic, Phoenix, AZ 85259, USA; ^8^Department of Internal Medicine, King Faisal Specialist Hospital and Research Center, 12713 Riyadh, Saudi Arabia

**Keywords:** ventricular tachycardia, catheter ablation, multimodality imaging, cardiac magnetic resonance, late gadolinium enhancement, multidetector computed tomography, intracardiac echocardiography, electroanatomical mapping, positron emission tomography, myocardial scar

## Abstract

Multimodality imaging plays a pivotal role in ventricular tachycardia (VT) ablation by providing critical insights into arrhythmogenic substrates and guiding procedural strategies. This updated review explores the integration of various imaging techniques, including echocardiography, cardiac magnetic resonance (CMR), multidetector computed tomography (MDCT), electroanatomical mapping (EAM), and nuclear imaging, to optimize VT ablation outcomes. Echocardiography, particularly transthoracic echocardiography (TTE), is an essential first-line tool for evaluating structural heart disease and left ventricular function. Moreover, echocardiography aids in risk stratification and procedural planning by detecting regional wall motion abnormalities and thrombus formation. Meanwhile, intracardiac echocardiography (ICE) enhances ablation precision by providing real-time catheter visualization, improving procedural success, and reducing complications such as cardiac tamponade. Nonetheless, CMR is the gold standard for myocardial tissue characterization, enabling the identification of scar burden and conduction channels critical for VT pathogenesis. Late gadolinium enhancement (LGE) facilitates preprocedural planning by localizing arrhythmogenic substrates, predicting VT recurrence risk, and informing ablation strategies. Additionally, T1- and T2-mapping techniques refine the assessment of myocardial fibrosis and inflammation, further improving patient selection and outcomes. MDCT complements CMR by offering high-resolution anatomical visualization and aids in delineating scar distribution, epicardial fat, and vascular structures, informing safe and effective ablation approaches. The integration of these imaging modalities significantly enhances VT ablation precision, reduces recurrence rates, and improves patient outcomes.

## 1. Introduction

Multimodality imaging has become essential for catheter ablation planning of ventricular tachycardia (VT), particularly in identifying and characterizing the arrhythmogenic substrate. The integration of various imaging modalities, such as cardiac magnetic resonance (CMR), multidetector computed tomography (MDCT), and electroanatomical mapping (EAM), has shown significant benefits in identifying these substrates, guiding VT ablation procedures, and improving overall clinical outcomes [1]. This review discusses the different imaging modalities, evidence gaps, and future directions in guiding VT ablation.

## 2. Echocardiography

Echocardiography can help risk-stratify patients by assessing parameters like left ventricular ejection fraction (LVEF) to determine the need for implantable cardioverter-defibrillator (ICD) therapy [2]. Echocardiography is the initial imaging modality for evaluating and managing VT. It is primarily used to assess the presence of structural heart disease, which is a common underlying cause of VT. According to the European Heart Rhythm Association (EHRA), Heart Rhythm Society (HRS), Asia Pacific Heart Rhythm Society (APHRS), and Latin American Heart Rhythm Society (LAHRS), echocardiography is recommended for evaluating ejection fraction (EF) and structural heart disease (SHD) in patients with VT [3]. It is favored due to its availability, cost-effectiveness, and the expertise available for its use. Transthoracic echocardiography (TTE) is typically performed to identify structural abnormalities such as left ventricular hypertrophy, dilated cardiomyopathy, or valvular heart disease, which can predispose patients to VT [4].

Additionally, TTE can be used to identify other causes of VT such as mitral annular disjunction, arrhythmogenic right ventricular cardiomyopathy, and hypertrophic cardiomyopathy. Echocardiography is an effective tool for detecting regional wall motion abnormalities, which are often linked to areas of low bipolar voltage. These areas may represent arrhythmogenic substrates crucial for ventricular tachycardia (VT) ablation. The American Society of Echocardiography (ASE) guidelines emphasize the use of 2D TTE for this purpose, noting that it provides comprehensive coverage of the left ventricle (LV) endocardial motion for all segments, although the apex may be poorly visualized due to foreshortening [5]. Enhanced echocardiography with contrast agents can improve visualization and diagnostic accuracy, particularly in technically challenging cases [6]. The detection of LV thrombus is crucial in patients undergoing VT ablation to prevent thromboembolic complications. The ASE guidelines recommend 2D TTE as the technique of choice for evaluating LV thrombus, with a sensitivity of 95% and specificity of 85–90% [7]. The use of echocardiographic contrast agents is particularly beneficial in cases where the apex is not clearly visualized, as it enhances the detection of thrombi by improving endocardial border definition and identifying filling defects [6]. However, it is important to note that while 2D TTE is effective, CMR has been shown to be more sensitive in detecting thrombi, particularly small or mural thrombi [8].

### Intracardiac Echocardiography

Intracardiac echocardiography (ICE) has also been utilized during VT ablation procedures to provide detailed imaging of the ventricular anatomy and to identify scar substrates that may be responsible for the arrhythmia. This technique enables precise mapping and targeting of ablation sites, reducing extensive fluoroscopy time [9]. The utility of ICE has been demonstrated for complicated anatomical sites such as challenging parahisian arrhythmias and ventricular arrhythmias originating from the papillary muscles [10, 11]. Furthermore, ICE in tandem with conventional EAM for VT ablation in patients with structural heart disease with a history of implantable cardiac device (ICD) and/or cardiac resynchronization therapy (CRT) has demonstrated a lower rate of 12-month VT-related readmission and repeat VT ablation compared to non-ICE patients (18.13% vs. 22.51%, *p* < 0.05; 14.35% vs. 19.34%, *p* = 0.02 respectively) although the cardiovascular (CV)-related readmissions and 12-month all-cause readmission rates were comparable between the two groups (44.56% vs. 43.20%, *p* = 0.62; 35.20% vs. 32.93%, *p* = 0.38) [12]. Additionally, ICE has been shown to improve the ability to locate and ablate the target isthmus in patients with repaired tetralogy of Fallot anatomy (ICE: 87% vs. no ICE 36%, *p* = 0.014). The recent SOUNDSCAR study results show a strong enough correlation with scars defined by ICE compared to electroanatomical mapping. The guidance by ICE showed a shorter procedural time with comparable VT-free survival in this small study compared to conventional EAM ablation [13].

ICE can also enhance ablation precision by allowing real-time visualization of the catheter position and the myocardial substrate, ensuring strong catheter-to-myocardial contact [14, 15]. For substrate identification, the operator utilizes intracardiac echocardiography to identify correlating regions of hyperechogenicity with wall thinning, hypo, or akinesia in the setting of ischemic cardiomyopathy or myocardial or subepicardial hyperechogenicity in nonischemic cases [16]. The increased ability to define anatomy with ICE can potentially obviate the need for adjunctive epicardial ablation in right ventricular free wall aneurysm cases. Furthermore, a non-fluoroscopic approach to VT ablation has been demonstrated utilizing ICE in combination with electroanatomical mapping for guidance [17]. An additional notable advantage of ICE is the early detection of complications such as pericardial effusion, thrombi, or steam pops. A recent study also demonstrated a lower prevalence of cardiac tamponade in patients undergoing VT ablation with intracardiac echocardiography [18]. Additionally, ICE provides real-time visualization of the catheter tip-tissue interface, which is crucial for assessing and maintaining optimal catheter contact and orientation. This capability enhances the precision of lesion formation by ensuring that the catheter is in the correct position and making adequate contact with the tissue, thus ensuring effective energy delivery [19]. Furthermore, ICE can identify inadequate catheter contact through the visualization of microbubbles, which are associated with smaller lesion volumes, thus allowing for immediate adjustments to improve energy delivery [19]. This real-time feedback is critical for achieving effective and safe ablation outcomes. Although ICE has been shown to improve VT-related outcomes in challenging patient groups, its cost-effectiveness in changing all-cause outcomes has not been demonstrated. The utility of ICE remains invaluable as it can provide critical information regarding ablation targets and precise anatomical details, especially in anatomically challenging cases.

## 3. Cardiac Magnetic Resonance (CMR) Imaging

### 3.1 Diagnosis

**Fig. 1.**
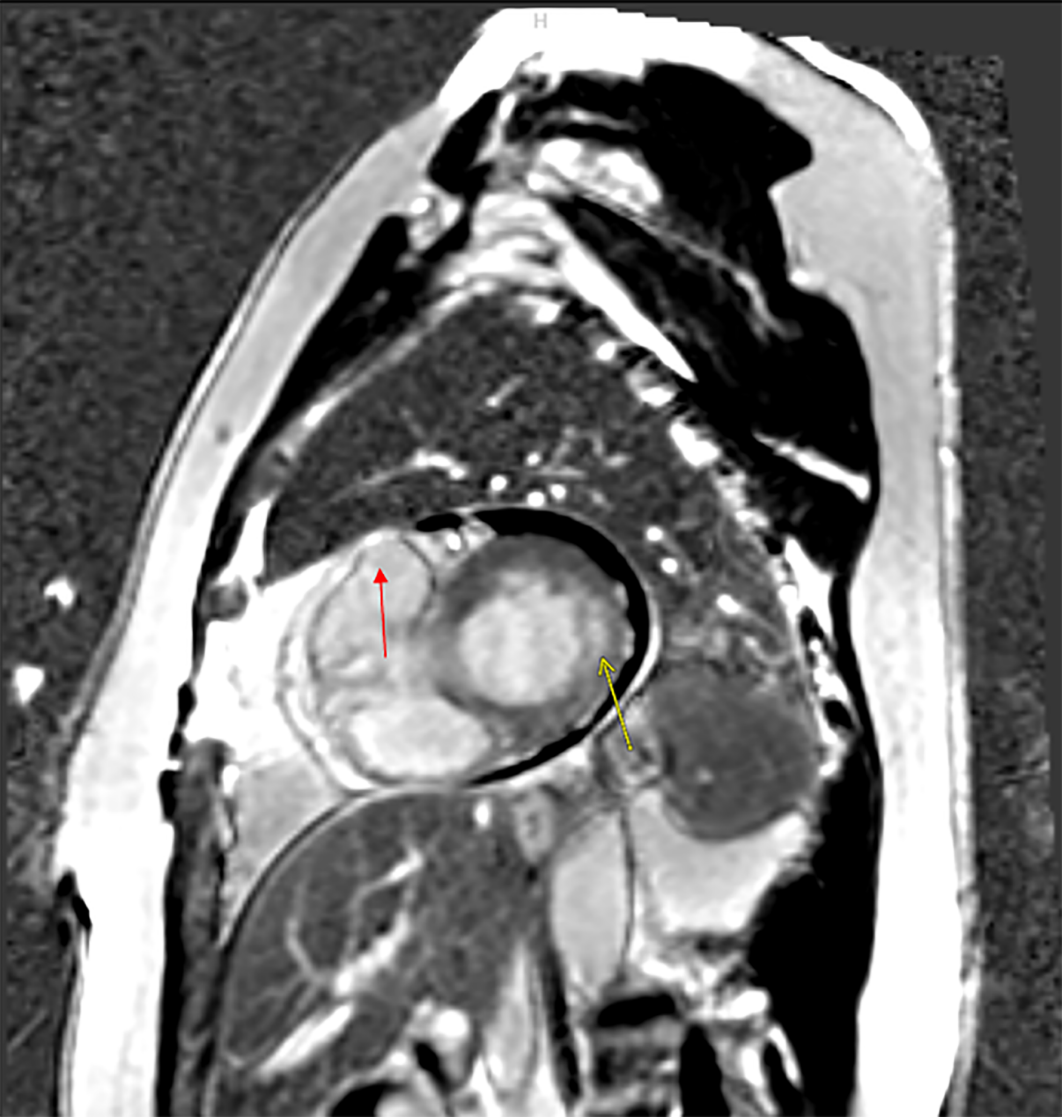
**Cardiac Magnetic Resonance Imaging (CMRI) images demonstrating 2 foci of delayed enhancement and vascular distributions consistent with 2 separate myocardial infarcts in a patient with ventricular tachycardia (VT)**. Infarct 1 (yellow arrow) is a small size, mild-severity subendocardial infarct involving the basilar lateral segment with slight extension into the anterolateral and inferolateral segments. It involves slightly less than 50% of myocardial thickness highly suggestive of residual viability. Infarct 2 (partially seen in red arrow) is a moderate-sized, severe transmural infarct involving the true apex and apical septal segments completely and portions of the anterior and inferior apical segments. While the edges of the infarct may be somewhat viable, the majority, probably at least 80%, of the infarct is transmural/nonviable.

**Fig. 2.**
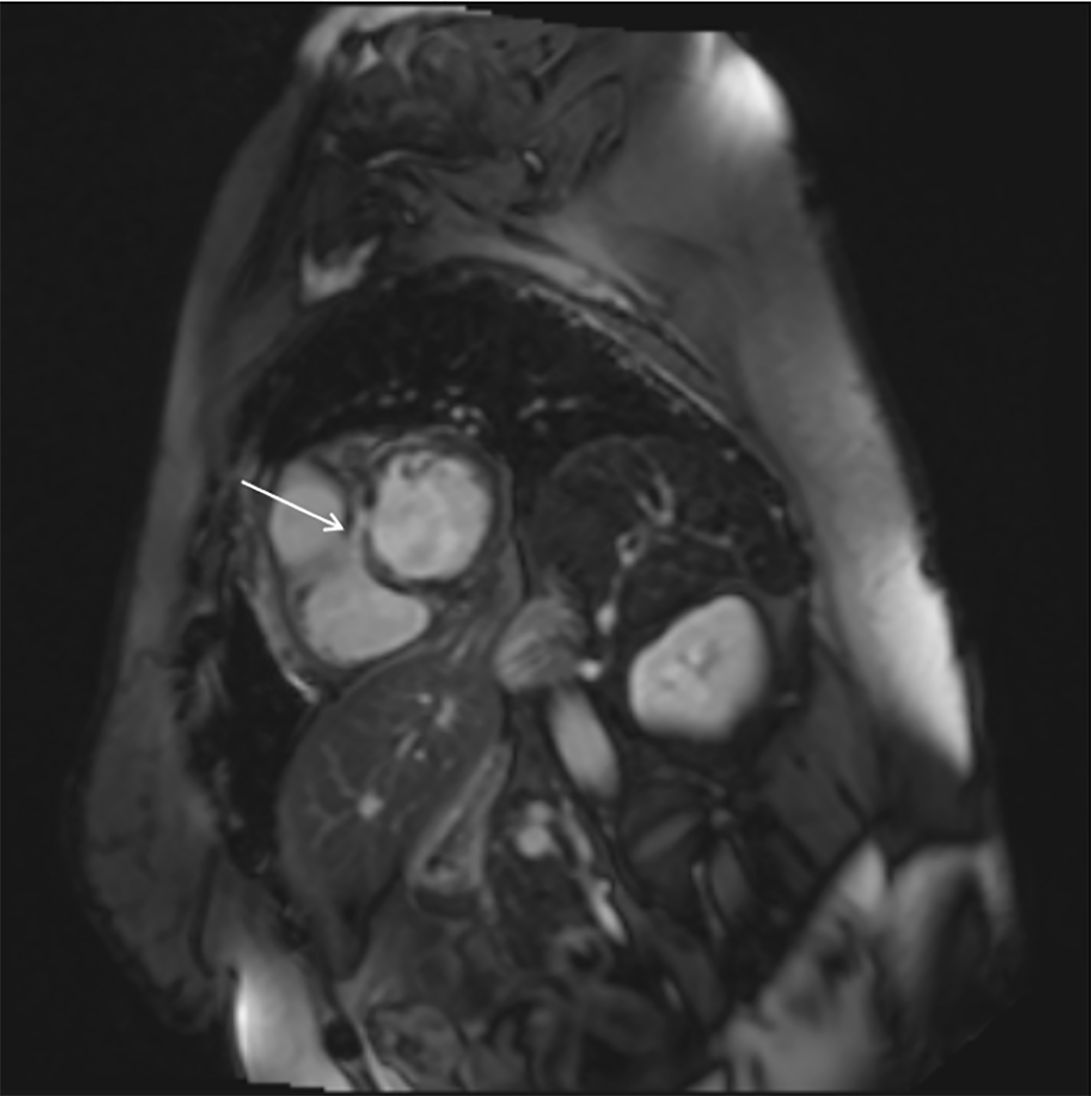
**CMRI image demonstrating patchy, multifocal linear areas of mid-myocardial and subepicardial delayed enhancement in a patient with myocarditis who presented with VT (white arrow)**.

Previous data suggested that CMR has significant diagnostic and prognostic value in VT (Figs. 1,2). CMR utilizes radiofrequency pulses and a powerful magnetic field to generate images of the heart [20]. It is considered the gold standard noninvasive test for detecting myocardial scar tissue, which is critical in the pathogenesis of VT [21, 22, 23]. It can detect intracardiac scars in 25% of apparently normal hearts [23]. The ability of CMR to differentiate between normal myocardial tissue and scar tissue is based on the relative differences in uptake and washout velocity of gadolinium contrast agents in myocardial tissue and scar tissue. With gadolinium lasting longer in the scarred tissue, selecting the correct timing to obtain late-acquisition images is essential in rendering the scarred tissue highlighted on T-1 weighted imaging while the normal myocardium is “blackened” or “nulled” (Fig. 1) [16, 20, 22].

Multiple studies have shown that ventricular tachycardia isthmus sites localize to areas of late gadolinium enhancement (LGE) in both ischemic cardiomyopathy (ICM) and non-ischemic cardiomyopathy (NICM) [16, 23]. Furthermore, the signal intensity of contrast uptake can quantify the volume of abnormal tissue and its transmurality, allowing for the identification of dense scar and the border zone [23]. Studies have shown that CMR can identify SHD in a substantial proportion of patients presenting with VT. For instance, Ge *et al*. [24] reported that CMR detected structurally abnormal hearts in 66% of patients with sustained VT or aborted sudden cardiac death (SCD). Moreover, T-1 mapping on cardiac magnetic resonance quantitatively identifies myocardial extracellular collagen [21, 23]. This allows for identifying myocardial scar tissue in patients with NICM and diffuse fibrotic disease, where the relative differences in uptake may not be high enough to allow areas of scar tissue to appear enhanced [23]. Utilizing this technique, Muser *et al*. [25] demonstrated that increase fibrosis detected through T1 mapping correlated with larger abnormal electrical circuits and worse long-term arrhythmia outcomes.

Late gadolinium enhancement cardiac magnetic resonance (LGE-CMR) is now the preferred diagnostic imaging modality in diagnosing arrhythmogenic ventricular cardiomyopathy (ARVC) [20]. Patients with ARVC display fibrofatty infiltration, mainly in the epicardial layers. Areas of DE-CMR identified scar tissue in ARVC are closely related to low electrogram (EGM) voltage and are highly specific for late-potential-containing areas in a subset of patients undergoing VT epicardial ablation. However, due to the mobility of the right ventricle and its thin wall, the imaging aspect remains a challenge. The acquisition of right ventricle strain imaging to identify contractile abnormalities due to right ventricular scar alongside LGE-CMR has been shown to improve the ability to localize VT target sites [21, 23].

### 3.2 Prognosis

The presence of LGE on CMR is associated with an increased risk of major adverse cardiac events (MACE) in patients with VT. Ge *et al*. [24] found that an abnormal CMR was associated with a higher annual rate of MACE in both non-sustained VT (NSVT) and sustained VT/aborted sudden cardiac death (SCD) cohorts. Additionally, Dawson *et al*. [26] demonstrated that fibrosis detected by LGE-CMR is an independent predictor of adverse outcomes, including cardiac death and appropriate ICD discharges [24]. Additionally, CMR can confer prognostic information in patients with scarred myocardium. In idiopathic dilated cardiomyopathy, the finding of mid-wall septal scarring with LGE predicts the occurrence of SCD and VT. In addition, patchy septal midwall enhancement is the classic finding in hypertrophic cardiomyopathy. The presence of a thin wall with decreased left ventricular (LV) function confers a poor 5-year prognosis. Similarly, the detection of aneurysms in apical hypertrophic cardiomyopathy has been associated with worse prognosis. The overall burden of contrast enhancement has also been associated with worse outcomes [20].

### 3.3 Procedural Planning and Success

Utilizing LGE-CMR in preprocedural planning improves outcomes in both ICM and NICM patients [21]. It can aid in candidate and approach selection in patients with epicardial and intramural scarring [21, 22]. Many patients with NICM and VT undergo an endo-epicardial approach adding to the duration and the risk of the procedure. LGE-CMR’s ability to identify epicardial fibrosis can aid in candidate selection who might benefit from an epicardial ablation and has been shown to improve outcomes [21, 22, 23]. Furthermore, LGE-CMR can identify epicardial scars that can be accessed through the coronary venous system (CVS) and may not require a percutaneous epicardial approach [23]. This is often seen in NICM patients with basal-predominant scars accessible through the CVS. As for intramural scars, a biventricular approach aided by LGE-CMR has been associated with better outcomes in NICM [22, 23].

In addition, LGE-CMR has been shown to aid in identifying patients who require adjunctive ablation measures, such as bipolar ablation, simultaneous unipolar ablation, and alcohol-based ablation [23]. LGE-CMR can further identify patients with fibrosis at the base of the papillary muscles and in inferolateral regions of the heart, two populations in which epicardial access may be necessary. Furthermore, CMR has prognostic implications. T-2 mapping on CMR can further help with preprocedural planning by planning the timing of ablations in patients with myocarditis and cardiac sarcoidosis (Fig. 2). [23] It can detect myocardial edema, aiding in the diagnosis of conditions like acute myocarditis and acute myocardial infarction [21]. Unlike chronic myocardial inflammation, T2 mapping can detect acute myocardial inflammation. During the acute phase of myocarditis and sarcoidosis, ablation has been associated with greater recurrence than in the chronic phase [23].

Additionally, CMR can identify critical isthmus sites and conduction channels essential for effective ablation. Piers *et al*. [27] showed that CMR-derived scar characteristics could guide the identification of critical isthmus sites during VT ablation. Moreover, Quinto *et al*. [28] highlighted that pre-procedural LGE-CMR could predict VT recurrence post-ablation by identifying factors such as septal involvement and transmural channels.

### 3.4 Intraprocedural Guidance

As for intraprocedural guidance, LGE-CMR can help focus catheter mapping to the scarred zone of the myocardium. It can also identify the depth and width of heterogeneous tissue channels (HTC). Through retrospective studies, the integration of LGE-CMR segmented scar was associated with better outcomes in patients with NICM. On the prospective side, EAMs focused in areas of LGE-CMR were associated with lower recurrence rates. Furthermore, the integration of CMR in VT ablations with a dechanneling technique was associated with a lower need for radiofrequency delivery, higher non-inducibility rates following ablation, and reduced recurrence of ventricular tachycardia [22]. VT recurrence or exacerbation post ablation is associated with poor long-term outcomes. This can happen due to incomplete targeting of the arrhythmic substrates or new scars formed through successful ablations. Following ablations, the myocardium can undergo changes, including acute inflammation and edema, transitioning to a chronic phase with microvascular obstruction and scar deposition. LGE-CMR can aid in identifying this newly formed scar tissue to guide repeat ablation procedures [23].

CMR can also assess arrhythmia substrate multidimensionally by recreating a 3D structure of conduction channels localized to the border zones of LGE-CMR. Conduction channels with an intramural or transmural path are associated with higher VT recurrence post ablation. Incorporating LGE-CMR into the ablation approach in patients undergoing scar dechanneling was associated with shorter procedure times and improved arrhythmia-free survival [16, 23].

### 3.5 Limitations

Despite its utility prior to VT ablation, CMR has certain limitations. Gadolinium-based agents have been associated with nephrogenic systemic fibrosis in patients with severe kidney dysfunction. However, with appropriate agent selection, the risk of NSF is thought to be very low (0.07%) even in patients with glomerular filtration rates below 30 mL/min/1.73 m^2^ [23]. DECMR has also been deemed safe for patients with implantable cardiac defibrillators and devices that have traditionally been considered contraindications [16]. Metal artifacts from defibrillators remain an issue, especially in areas of the heart that are close to the pulse generators and leads, such as the anterior and apical segments [23]. With the use of optimized imaging sequencing and modified wideband inversion recovery technique, mild to moderate metal artifacts can be eliminated [25]. Contraindications to CMR include ferromagnetic implants such as certain older cerebrovascular clips, patients with severe allergies to gadolinium, or high-risk medical conditions such as hemodynamically unstable patients, those with respiratory compromise, or other critical illnesses that may preclude safe magnetic resonance imaging (MRI) planning. Ultimately, CMR’s detailed viable and tissue scar characterization can assist in preprocedural planning and intraprocedural guidance, allowing for targeted ablation strategies.

## 4. Cardiac Computed Tomography (CCT)

**Fig. 3.**
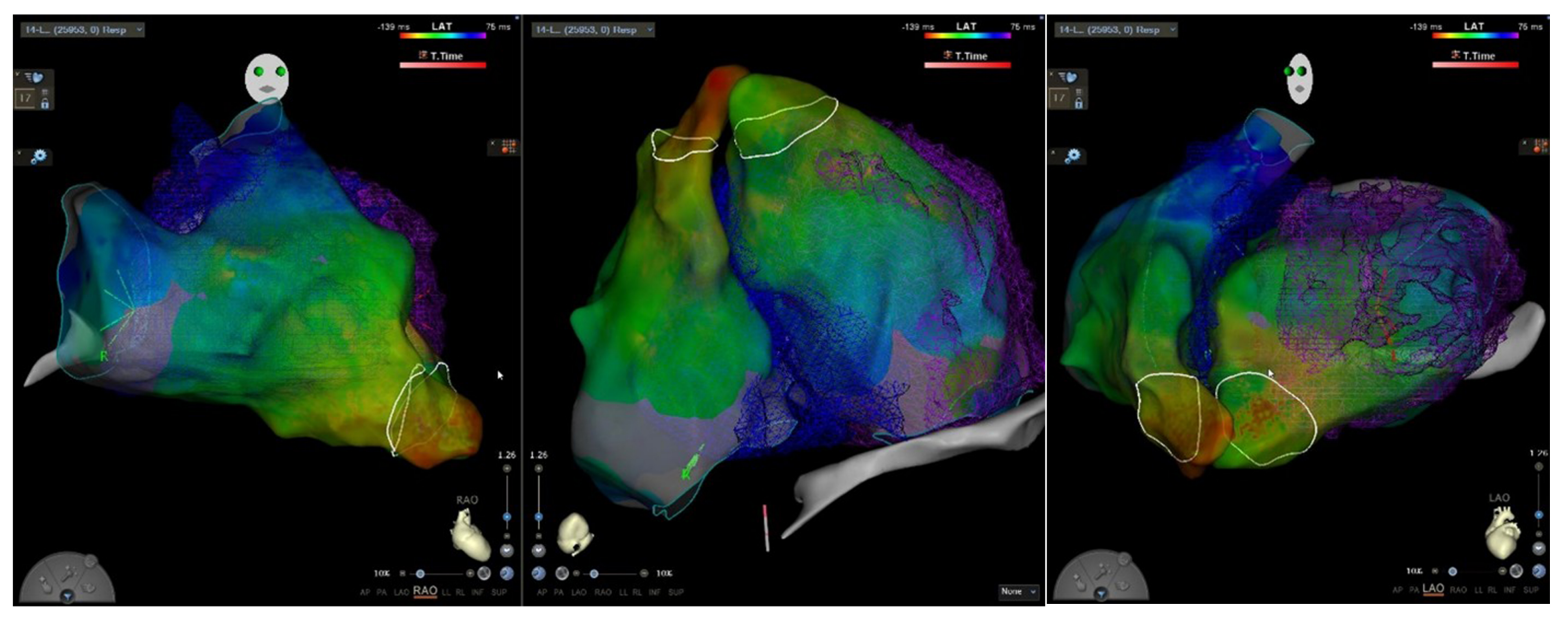
**Ablation procedure aided by multidetector cardiac computed tomography (MDCT) integration in a patient with ventricular tachycardia**.

Cardiac Computed Tomography (CCT) has been increasingly utilized to assess and plan VT ablations, particularly in cases where CMR is contraindicated (Fig. 3). CCT offers several advantages, including superior spatial resolution compared to CMR, allowing for a more detailed evaluation of myocardial structure and function. However, a key limitation of CCT is its lower contrast-to-noise ratio, which limits its ability to characterize myocardial scar tissue [22]. Several methods for myocardial scar characterization using CCT have been described, including assessing myocardial wall thinning (MWT) and delayed iodine enhancement. MWT is frequently observed in regions of prior myocardial infarction, resulting from interstitial remodeling and cellular loss [29]. MWT has been shown to correspond with low-voltage regions and the distribution of local abnormal ventricular activities in patients with post-infarction VT [29]. However, while MWT is frequently observed in ischemic cardiomyopathy, it is less prevalent in non-ischemic cardiomyopathy, challenging its utility in this patient population [30].

CCT can also identify regions of preserved wall thickness within severely thinned myocardium, suggesting the presence of viable tissue within dense scar. These regions may serve as conduction channels, exhibiting a higher density of late potentials and increased VT risk [16]. Comparative analyses of CCT and EAM in detecting fibrosis and VT substrate have yielded encouraging results. Esposito *et al*. [31] also assessed the role of CCT and delayed enhancement in characterizing VT structural substrates. Their study showed that CCT effectively identified myocardial scars with a sensitivity of 76% and specificity of 86%, demonstrating a strong correlation with low-voltage regions on EAM [31]. Carbucicchio *et al*. [32] reported that CCT-based preprocedural assessment before VT ablation is a viable approach, demonstrating a strong correlation with EAM in detecting myocardial fibrosis, with an overall Cohen’s κ of 0.933. However, these studies are limited by small sample sizes and specific patient populations, which limits the generalizability of their findings.

CCT’s enhanced spatial resolution allows for a detailed assessment of anatomical structures, including the coronary vasculature and phrenic nerves. This is particularly useful in the procedural planning of epicardial ablations, allowing for the identification of unsafe ablation sites and minimizing the risk of complications involving adjacent structures (Fig. 3). CCT also improves the characterization and quantification of epicardial fat while also distinguishing it from myocardial scar, both of which exhibit low voltages on EAM [33]. Lastly, Alyesh *et al*. [34] demonstrated that myocardial calcifications detected by CCT in postinfarction patients are associated with VT. These calcifications represent areas of electrical nonexcitability and form boundaries for reentry circuits, which are crucial for effective ablation [34]. While CCT provides several advantages, CMR remains the gold-standard non-invasive imaging modality for identifying VT substrates. However, CCT is a valuable adjunct, offering detailed anatomical visualization and enhancing procedural guidance. Ongoing developments in CCT technology, coupled with the integration of artificial intelligence, may further strengthen its role in risk stratification and procedural planning for complex ablations.

## 5. Nuclear Imaging

Nuclear imaging plays a significant role in assessing and managing VT, particularly in identifying the arrhythmogenic substrate and guiding ablation therapy. While not routinely used for all VT ablation cases, it can provide detailed insights into the metabolic pattern, active inflammation, or ischemia characteristics of VT substrates, aiding in identifying ablation targets and potentially improving clinical outcomes in patients with VT. For example, in active inflammation and myocardial viability with ^18^F-fluorodeoxyglucose (^18^F-FDG), myocardial perfusion can be assessed by 11C-acetate, 13N-ammonia, 15O-water, and 82-Rubidium (Rb), and sympathetic innervation assessment through the use of an analog of norepinephrine such as (^123^I-MIBG) and 11C-hydroxyephedrine (11C-HED).

### 5.1 Positron Emission Tomography (PET)

**Fig. 4.**
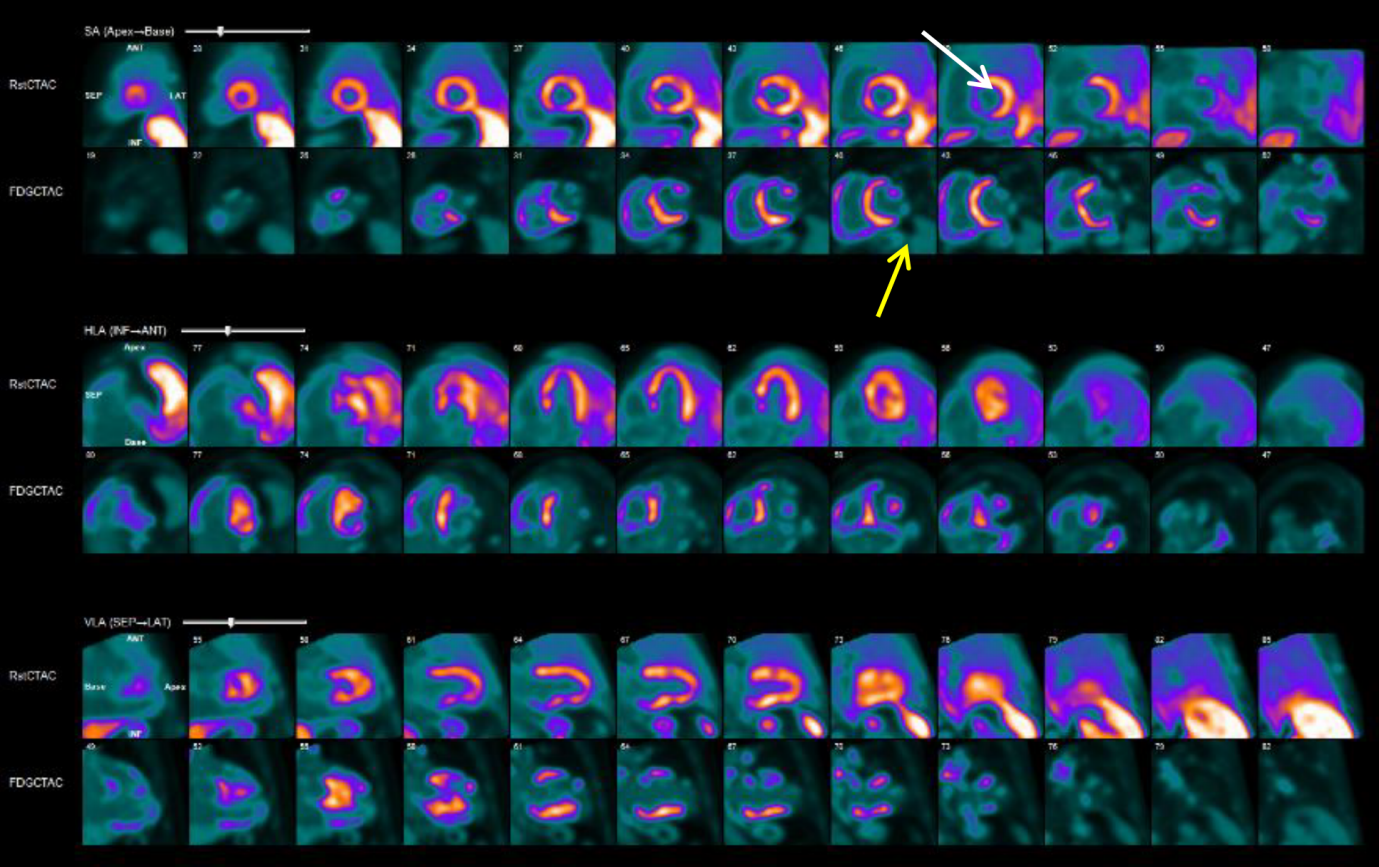
**N-13 ammonia imaging in a patient with cardiac sarcoidosis and VT**. The figure demonstrates foci of decreased activity at the inferior wall from mid ventricle to base, anteroseptal wall at the base, and anterolateral wall at the base (white arrow). On F-18 FDG imaging, there are uptake foci at the anteroseptum, inferoseptum, septum, and inferior walls from mid-ventricle to base and anterior/anterolateral walls at the base, which are suspicious for myocardial inflammation (yellow arrow).

PET is critical in identifying and characterizing arrhythmogenic substrates, especially in conditions like sarcoidosis and myocarditis [35]. It uses specific tracers to detect abnormal myocardial perfusion, active inflammation, evaluation of sympathetic innervation, or myocardial viability, all of which contribute to arrhythmogenic activity [36]. This modality assesses metabolic scar tissue in patients with ischemic cardiomyopathy undergoing VT ablation (Fig. 4). Studies have shown that areas with severe PET defects (defined as <50% uptake) correlate with regions of dense scarring and abnormal voltage on electroanatomic maps (EAMs). These metabolic abnormalities are predictive of VT channels or exit sites in approximately 90% of cases, making PET a valuable tool for identifying targets for ablation [37]. PET imaging can quantify myocardial blood flow, with the O-Water tracer being the gold standard for flow quantification since it is freely diffusible and metabolically inert [11]. 18 FDG-PET can also identify myocardial tissue viability [35] and inducible ischemia and aids in characterizing arrhythmogenic substrates before ablation [38].

### 5.2 Role of PET in Inflammation

F-FDG-PET is the gold standard for evaluating myocardial inflammation [39]. A prospective study by Tung *et al*. [40] evaluated 107 patients who experienced frequent premature ventricular contractions (PVCs) without apparent cause and underwent ^18^F-FDG PET scans, revealing that 51% had abnormal ^18^F-FDG uptake. Among these cases, 46% were linked to an underlying lymphocytic infiltrate, and 7% were associated with cardiac sarcoidosis in those with endomyocardial biopsies (EMB). Interestingly, after receiving immunosuppressive treatment, 82% of these patients saw at least a partial reduction in their arrhythmic burden [40]. This outcome highlights the crucial role of PET imaging in detecting myocardial inflammation (Fig. 4).

PET also has an important prognostic value in predicting VT recurrence in patients with sarcoidosis. In this population, patients with baseline positive PET scans had a fourfold higher risk of VT recurrence post-ablation, and those without PET improvement after immunosuppressive therapy had an additional twofold increased risk [41]. Moreover, ^123^I-MIBG identifies cardiac denervation and autonomic dysfunction in cardiac amyloidosis [42]. Tracers like C-HED can assess cardiac sympathetic activity, influencing VT onset and maintenance [43]. Therefore, detecting abnormal innervation areas correlates with effective catheter ablation targets, especially in ischemic cardiomyopathy (ICM).

Overall, PET is crucial in detecting myocardial inflammation, differentiating scar vs. active disease, predicting VT recurrence, guiding therapy, and identifying optimal ablation targets in inflammatory cardiomyopathies and VT. Its integration with other imaging modalities enhances procedural planning and improves the targeting of arrhythmogenic areas during VT ablation.

### 5.3 I-Metaiodobenzylguanidine (MIBG) SPECT Imaging

MIBG SPECT is used to evaluate cardiac sympathetic innervation. Abnormal innervation, as indicated by reduced MIBG uptake, has been associated with VT substrate. Studies have demonstrated that abnormal innervation areas often overlap with scar regions identified by cardiac MRI and low-voltage areas on EAMs. ^123^I-MIBG imaging helps create three-dimensional innervation maps, showing that areas of denervation are more extensive than those defined by bipolar voltage criteria. While studies have demonstrated that ^123^I-MIBG may overestimate EGM-defined scar areas, successful ablation sites often fall within these denervated areas. This suggests that ^123^I-MIBG imaging can provide critical information about the arrhythmogenic substrate and improve the precision of VT ablation, especially when used in conjunction with ^18^F-FDG PET [44]. This multimodality approach helps identify heterogeneous adaptations within the VT substrate that are not fully appreciated by any imaging modality alone [45, 46]. For example, PET is effective in diagnosing CA, while ^123^I-MIBG can detect cardiac denervation and autonomic dysfunction in this patient population [43]. As a lone imaging modality, ^123^I-MIBG SPECT has demonstrated a role in heart failure, predicting VT inducibility and ICD shocks in these patients.

## 6. Multimodality Imaging Integration

Integrating data from multiple imaging modalities with electroanatomic mapping systems has become increasingly prevalent. This integration allows for a comprehensive assessment of the arrhythmogenic substrate, combining anatomical, functional, and electrophysiological information. Such a comprehensive approach facilitates more effective and safer ablation procedures by providing precise targets and minimizing collateral damage to surrounding healthy tissue, resulting in improved procedural outcomes (Table 1).

**Table 1.** **Summary of multimodality imaging modalities for VT ablation**.

Imaging modality	Benefits	Limitations
Intracardiac echocardiography	■ Real-time assessment of cardiac structure and function	■ Limited field of view, making it challenging to visualize the entire left ventricle or epicardial structures comprehensively
	■ Intraprocedural guidance	■ Operator dependent
	■ Facilitating transeptal access	■ Invasive, requiring additional venous access
	■ Optimizing catheter-tissue endocardial contact	■ Cost-effectiveness in changing outcomes is limited
	■ Rapid evaluation of procedural complications	
Multidimensional computed tomography	■ Provides detailed assessment of coronary vasculature to guide pre-procedural planning	■ Low contrast-to-noise ratio, reducing the capability to distinguish between normal myocardium and scar
	■ Detection of myocardial scar and distinguishing it from epicardial fat regions	■ Inability to differentiate between scar and channels using myocardial wall thickness
		■ Increasing radiation exposure to patients
Cardiac magnetic resonance	■ Assessment of endocardial, midmyocardial, and epicardial scar	■ Low spatial resolution that may be insufficient to detect small bundles of myocytes with potential for reentry
	■ Evaluation of remodeling and myocardial extracellular space expansion	■ Metal artifacts from defibrillators
		■ Long acquisition times
		■ Need for optimization of high resolution, 3D tissue characterization
Nuclear imaging	■ Provides detailed insights into metabolic patterns, active inflammation, and ischemia characteristics of VT substrates	■ Limited spatial resolution compared to MDCT or CMR
	■ Identification of myocardial tissue viability and inducible ischemia	■ Exposure to ionizing radiation
	■ Aids in identifying optimal ablation targets	■ Technically challenging to integrate data with electroanatomical mapping (EAM) systems
		■ Cost

Studies have demonstrated that imaging-derived substrates can accurately identify critical VT isthmuses and local abnormal ventricular activities (LAVA). Yamashita *et al*. [47] reported that imaging-derived substrates identified 89% of critical VT isthmuses and 85% of LAVA, with higher efficiency in ICM and arrhythmogenic right ventricular cardiomyopathy (ARVC) compared to NICM. This integration also influenced procedural management, motivating additional mapping and epicardial access in many patients. Furthermore, Berruezo *et al*. [22] highlighted that integrating LGE-CMR or MDCT into the navigation system has become a cornerstone for VT ablation in some centers of excellence. This integration aids in complete substrate identification and characterization, focusing EAM on regions of interest, thereby improving procedural efficiency and outcomes [22]. Additionally, real-time image integration has been shown to be an independent predictor of VT-free survival post-ablation. Yamashita *et al*. [48] found that real-time image integration, achieving complete LAVA elimination, and multipolar catheters were significant predictors of better outcomes.

### Timing and Workflow of Imaging Integration in VT Ablation

In contemporary VT ablation practice, multimodality imaging is most effective when applied within a clearly defined temporal workflow that includes pre-procedural planning, intraprocedural guidance, and post-procedural reassessment [22]. Pre-procedurally, LGE-CMR is commonly obtained days to weeks before ablation to characterize myocardial scar location, transmural extent, and the presence of heterogeneous tissue channels, particularly in patients with ischemic and non-ischemic cardiomyopathy. When CMR is contraindicated, MDCT can provide an alternative means of substrate and anatomical assessment through evaluation of myocardial wall thinning and delayed iodine enhancement. In patients with suspected inflammatory cardiomyopathies, ^18^F-FDG PET is typically performed prior to ablation to assess disease activity and to inform both immunosuppressive therapy and procedural timing [20, 23].

During the ablation procedure, previously acquired CMR or CT datasets are frequently segmented and integrated into electroanatomical mapping systems. This approach allows mapping efforts to be focused on predefined regions of interest, facilitates identification of potential critical isthmuses, and may reduce overall mapping time. Intracardiac echocardiography serves an important complementary role by providing real-time visualization of catheter position, tissue contact, scar morphology, and pericardial space, thereby improving procedural safety and decision-making [9].

Following ablation, repeat imaging—most often with CMR—may be considered in selected patients with VT recurrence to assess for residual arrhythmogenic substrate or newly formed scar. Adoption of a consistent imaging workflow across these procedural phases may improve reproducibility and outcomes across centers [20, 22].

From a practical clinical perspective, the available imaging modalities should be considered complementary rather than interchangeable. CMR offers the most detailed assessment of myocardial substrate and is generally preferred when feasible, particularly in non-ischemic cardiomyopathy. MDCT is especially useful in patients unable to undergo CMR and in cases where detailed delineation of coronary anatomy, epicardial structures, or phrenic nerve course is required for procedural planning. Intracardiac echocardiography plays a key intraprocedural role by enhancing safety, optimizing catheter-tissue contact, and providing real-time anatomical feedback. Nuclear imaging techniques are most informative in inflammatory or infiltrative cardiomyopathies, where assessment of disease activity directly influences both the timing and strategy of VT ablation. Taken together, a tailored, multimodality imaging approach allows VT ablation strategies to be individualized to the underlying pathology and clinical context.

## 7. Clinical Outcomes

There are no published randomized controlled trials that directly compare imaging-guided ablation to conventional ablation for ventricular tachycardia in terms of long-term clinical outcomes. A pilot study of CT-guided VT ablation targeting wall thickness heterogeneity achieved 61.9% VT-free survival at mean follow-up of 47.8 months [49]. However, a comparison of ultra-high-density mapping versus conventional point-by-point mapping found no significant difference in long-term VT-free survival or shock-free survival [50]. Multiple cohort studies have shown that pre-procedural LGE-CMR integration is associated with lower VT recurrence rates and improved arrhythmia-free survival compared with conventional substrate mapping alone, with recurrence reductions of approximately 25–40%, particularly in non-ischemic cardiomyopathy [27]. In inflammatory cardiomyopathies, PET-guided identification of active inflammation and subsequent immunosuppressive therapy prior to ablation has been shown to significantly reduce VT burden and improve long-term arrhythmia control [41]. Ongoing trials such as STABLE-VT and the PET/MR evaluation study will provide critical data on imaging-aided VT ablation outcomes.

## 8. Emerging Technologies

Technological advances aim to improve the precision and outcomes of VT ablation by providing detailed and accurate mapping of arrhythmogenic substrates, predicting optimal ablation targets, potentially reducing recurrence rates, and improving long-term outcomes.

**(1). Omnipolar Mapping:** This technology generates electroanatomic voltage maps with orientation-independent electrograms, providing higher point density and more accurate identification of late potentials and isochronal crowding. It has shown higher specificity in detecting VT isthmuses compared to traditional bipolar mapping [51].

**(2). High-Resolution Mapping Systems:** Systems like the Rhythmia mapping system use a minibasket 64-electrode catheter to create ultra-high-density maps rapidly and safely. This system has demonstrated reliable automatic annotation of VT circuits and consistent recording of abnormal electrograms [52].

**(3). CMR and CTT:** These imaging modalities are increasingly used for arrhythmogenic substrate identification and characterization. Integrating pixel signal intensity maps from LGE-CMR or wall thickness maps from MDCT into the navigation system enhances pre-procedural planning and procedural guidance [22, 53].

**(4). Functional Substrate Mapping:** This approach focuses on identifying critical isthmuses without requiring hemodynamic stabilization during VT, shifting the emphasis to analyzing potentials during baseline rhythm. This method addresses conduction abnormalities and repolarization heterogeneity [54].

**(5). Personalized Virtual-Heart Technology:** This technology uses cardiac imaging and computational modeling to identify optimal infarct-related VT ablation targets. It has shown promise in improving the accuracy of ablation target identification and reducing the extent of lesions [55].

**(6). Advanced Imaging Integration:** Combining CMR and MDCT with EAM systems allows for better substrate definition, particularly in defining border zones, tissue channels, and fat, thus enhancing the effectiveness and safety of VT ablation procedures [56].

These advancements aim to improve the precision and outcomes of VT ablation by providing detailed and accurate mapping of arrhythmogenic substrates.

## 9. Evidence Gaps and Future Directions

Despite the advancements in multimodality imaging for VT ablation, several evidence gaps persist:

**(1). Standardization of Imaging Protocols:** Currently, there are no standardized protocols for acquiring and interpreting imaging data in the context of VT ablation, and there is a limited understanding of combining different imaging techniques to assess the myocardial substrate in VF patients comprehensively. The European Heart Rhythm Association (EHRA), HRS, APHRS, and LAHRS highlight the need for further research to determine the optimal use of these modalities in risk stratification and treatment planning. Establishing uniform guidelines is essential to ensure reproducibility and comparability across different centers [3]. Standardized multimodality imaging protocols for ventricular fibrillation (VF) are required to improve risk prediction and procedural planning to improve overall outcomes [21, 57].

**(2). Long-Term Outcomes:** While short-term benefits of multimodality imaging-guided ablation have been demonstrated, data on long-term outcomes remain limited. Prospective studies with extended follow-up periods are necessary to validate the prognostic value of advanced imaging modalities to identify myocardial scar tissue associated with higher VF risk. The American College of Cardiology (ACC), American Heart Association (AHA), and HRS focus on the importance of studying novel imaging modalities and their integration to improve patient selection for ICDs [58].

**(3). Cost-Effectiveness Analysis:** The cost-effectiveness of implementing multimodality imaging in routine clinical practice has not been evaluated. Comprehensive cost-effectiveness analyses are needed to justify these technologies’ widespread adoption and inform healthcare policy decisions [21].

To address these gaps, future research directions should focus on the following:

### 9.1 Integration of Artificial Intelligence (AI) 

AI can play a significant role in the ablation of VT by enhancing the precision and efficiency of the procedure. AI techniques can be utilized in various areas:

**(1). VT Localization and Mapping:** AI algorithms, such as convolutional neural networks (CNNs), can analyze electrograms (EGMs) from cardiac implantable electronic devices (CIEDs) to localize the sources of focal VTs with high accuracy. This approach reduces the invasiveness and time required for traditional mapping techniques [59].

**(2). Computational Modeling:** AI-driven computational models, like the Virtual Induction and Treatment of Arrhythmias (VITA), can simulate VT induction and identify ablation targets in near real-time. These models use reaction-Eikonal methodology to detect scar-related VTs and compute ablation targets efficiently, significantly reducing the computational resources and time compared to traditional methods [59].

**(3). Personalized Virtual-Heart Technology:** This technology integrates cardiac imaging and computational modeling to create patient-specific virtual hearts. These models can predict optimal ablation targets, potentially improving the accuracy and outcomes of VT ablation procedures [55].

**(4). Procedure Efficiency:** AI-based arrhythmia mapping systems can reduce the time to first ablation, overall procedure duration, and fluoroscopy use, thereby enhancing procedural efficiency and patient safety [60].

### 9.2 Development of Noninvasive Ablation Techniques

Recent studies have shown promising results from emerging noninvasive techniques for VT ablation, such as stereotactic body radiation therapy (SBRT) utilizing multimodality imaging to deliver targeted radiation therapy to arrhythmogenic foci, offering a potential alternative for patients who are poor candidates for traditional catheter-based ablation. Previous studies showed that SBRT significantly reduced VT episodes in patients with refractory VT or PVC-related cardiomyopathy. The median episode dropped from 119 to 3, and a 75% burden reduction was seen in 89% of patients, improving the quality of life with modest short-term risks [61]. Similarly, another study of five high-risk refractory VT patients treated with SBRT reported a 99.9% reduction in VT episodes, preserved left ventricular function, and mild, resolving lung inflammation within a year [62].

**(1). Personalized Medicine Approaches**: Precision or personalized medicine approaches in VT ablation have shown promising results in improving the identification of ablation targets and potentially enhancing procedural outcomes. Personalized heart digital twin technology uses cardiac imaging to create patient-specific heart models for simulating VT circuits and predicting optimal ablation sites. Studies show this approach improves substrate-based VT ablation by identifying regions with abnormal electrograms and conduction slowing [63]. Similarly, virtual-heart technology combines imaging and computational modeling to enhance infarct-related VT ablation target accuracy in retrospective and prospective studies [55]. Additionally, fast electrophysiological models derived from CT images simulate VT activation patterns and predict targets in a clinically feasible timeframe, potentially shortening procedures and improving outcomes [64].

**(2). Multicenter Collaborative Studies**: Large-scale multicenter studies are required to address the current gaps. Collaborative research efforts can provide robust data on the efficacy, safety, and long-term outcomes of multimodality imaging-guided VT ablation, determining best practices and guiding future directions.

## 10. Conclusion

Integrating multimodality imaging into VT ablation represents a significant advancement in managing ventricular arrhythmias. By providing detailed insights into myocardial structure and function, these imaging techniques enhance the precision of ablation procedures, leading to improved clinical outcomes. Addressing current evidence gaps through standardized protocols, long-term studies, and cost-effectiveness analyses is essential. Future advancements, particularly the integration of AI, the development of noninvasive techniques, and personalized medicine approaches, have the potential to improve patient outcomes in VT management even further.

## Abbreviations

2D, Two-dimensional; 3D, Three-dimensional; ACC, American College of Cardiology; AHA, American Heart Association; AI, Artificial Intelligence; APHRS, Asia Pacific Heart Rhythm Society; ARVC, Arrhythmogenic Right Ventricular Cardiomyopathy; ASE, American Society of Echocardiography; CA, Cardiac Amyloidosis; CCT, Cardiac Computed Tomography; CIED, Cardiac Implantable Electronic Device; CMR, Cardiac Magnetic Resonance; CNN, Convolutional Neural Network; CRT, Cardiac Resynchronization Therapy; CT, Computed Tomography; CV, Cardiovascular; CVS, Coronary Venous System; DE-CMR, Delayed-Enhancement Cardiac Magnetic Resonance; EAM, Electroanatomical Mapping; EF, Ejection Fraction; EGM, Electrogram; EHRA, European Heart Rhythm Association; EMB, Endomyocardial Biopsy; FDG, Fluorodeoxyglucose (typically ^18^F-FDG); GFR, Glomerular Filtration Rate; HRS, Heart Rhythm Society; HTC, Heterogeneous Tissue Channels; ICD, Implantable Cardioverter-Defibrillator; ICE, Intracardiac Echocardiography; ICM, Ischemic Cardiomyopathy; LAHRS, Latin American Heart Rhythm Society; LAVA, Local Abnormal Ventricular Activities; LGE, Late Gadolinium Enhancement; LV, Left Ventricle/Left Ventricular; LVEF, Left Ventricular Ejection Fraction; MACE, Major Adverse Cardiac Events; MDCT, Multidetector Computed Tomography; MIBG, Meta-Iodobenzylguanidine (e.g., ^123^I-MIBG); MRI, Magnetic Resonance Imaging; MWT, Myocardial Wall Thinning; NICM, Non-Ischemic Cardiomyopathy; NSF, Nephrogenic Systemic Fibrosis; NSVT, Non-Sustained Ventricular Tachycardia; PET, Positron Emission Tomography; PVC, Premature Ventricular Complex(es); Rb, Rubidium-82 (^82^Rb); SCD, Sudden Cardiac Death; SBRT, Stereotactic Body Radiation Therapy; SPECT, Single-Photon Emission Computed Tomography; T1, Longitudinal Relaxation Time (T1 Mapping); T2, Transverse Relaxation Time (T2 Mapping); TTE, Transthoracic Echocardiography; VF, Ventricular Fibrillation; VITA, Virtual Induction and Treatment of Arrhythmias (computational model); VT, Ventricular Tachycardia.
